# Physical Activity Intervention for Urban Black Women With Asthma: Protocol for a Randomized Controlled Efficacy Study

**DOI:** 10.2196/55700

**Published:** 2024-02-07

**Authors:** Ellen Davis, Elizabeth Townsend, Aero Cavalier, Yi-Fan Chen, Dameka Edwards-Hart, Spyros Kitsiou, Wiktoria Kowalczyk, Iliana Mansur, Ebere Okpara, Karen Powell, Valerie G Press, Toni Ramirez, Deborah Salvo, Lisa K Sharp, Brittani Wright, Sharmilee Maria Nyenhuis

**Affiliations:** 1 Section of Allergy and Immunology Department of Pediatrics University of Chicago Chicago, IL United States; 2 Center for Research on Health Care Data Center Department of Medicine University of Pittsburgh Pittsburgh, PA United States; 3 Department of Biomedical and Health Information Sciences University of Illinois Chicago Chicago, IL United States; 4 Department of Pharmacy, Systems, Outcomes and Policy University of Illinois Chicago Chicago, IL United States; 5 Section of General Internal Medicine Department of Medicine University of Chicago Chicago, IL United States; 6 People, Health and Place Lab Department of Kinesiology and Health Educations University of Texas Austin Austin, TX United States; 7 Department of Biobehavioral Nursing Science University of Illinois Chicago Chicago, IL United States; 8 Office of Research Facilitation College of Nursing University of Illinois Chicago Chicago, IL United States

**Keywords:** asthma, physical activity, lifestyle, Black women

## Abstract

**Background:**

Black women experience a higher prevalence of poor asthma outcomes and physical inactivity than their White counterparts. Black women comprise a particularly vulnerable group of patients with asthma, with some of the highest rates of asthma in adults, high health care use (emergency department visits and hospitalizations), and the highest crude asthma mortality rate of all race or ethnicity groups. Despite recommendations to engage in regular physical activity, fewer than 15% of Black women meet the 2008 National Physical Activity Guidelines, the lowest of all racial subgroups of adults. Given the connection between physical inactivity and poor asthma outcomes, addressing physical activity among Black women with asthma is imperative.

**Objective:**

This 2-arm randomized controlled trial aims to (1) determine the efficacy of a lifestyle walking intervention on asthma control compared to an education (control) group over 24 weeks, (2) examine the maintenance effects of the lifestyle walking intervention on asthma control at 48 weeks, (3) explore the behavioral mediators (eg, self-efficacy, social support, self-regulation, and daily physical activity levels) and contextual moderators (eg, baseline asthma severity, neighborhood environment, comorbid conditions, and social determinants of health) that contribute to treatment responsiveness, and (4) assess the reach and implementation potential of the intervention.

**Methods:**

The proposed study (ACTION [A Lifestyle Physical Activity Intervention for Minority Women with Asthma]) delivers a 24-week lifestyle walking intervention designed for and by urban Black women with asthma. Participants (n=224) will be recruited through 2 urban health care systems that care for a diverse Black population. Patients will be randomized to one of two groups: (1) ACTION intervention (group sessions, physical activity self-monitoring—Fitbit, and text-based support for step goal setting) or (2) education control (an individual asthma education session and SMS text messages related to asthma education). Outcome assessments will take place at baseline, 12, 24, and 48 weeks. The primary outcome is a change in asthma control from baseline to week 24 as assessed by the asthma control questionnaire-6 (ACQ-6). Secondary outcomes include asthma-related quality of life, health care use, and asthma exacerbations and behavioral outcomes such as self-efficacy, self-regulation, social support, and physical activity.

**Results:**

This study was funded by the National Institute of Minority Health Disparities in August 2022. We pilot-tested our recruitment and intervention procedures and began recruitment in April 2023, with the enrollment of our first participant in May 2023. The anticipated completion of the study is April 2027.

**Conclusions:**

This study will deliver a new approach to physical activity interventions in Black women with asthma and help to provide guidance for addressing physical activity within this subgroup. This study will also provide a potential framework for future studies in minoritized populations with other disease conditions associated with low levels of physical activity.

**Trial Registration:**

ClinicalTrials.gov NCT05726487; https://clinicaltrials.gov/study/NCT05726487

**International Registered Report Identifier (IRRID):**

DERR1-10.2196/55700

## Introduction

### Background

Asthma is a serious global health problem affecting about 358 million people worldwide [1]. Black women comprise a particularly vulnerable group of patients with asthma, with the highest rates of asthma among adults (11.4%), high health care use (emergency department visits and hospitalizations), and the highest crude asthma mortality rate of all racial or ethnic groups [2,3]. There are multiple factors that contribute to this increased morbidity and mortality including, high rates of exposure to environmental pollution, and limited access to quality health care [4]. Low levels of physical activity (PA) are associated with worse asthma outcomes including higher health care use, poorer lung function, lower asthma control, and decreased exercise capacity [2]. Despite recommendations to engage in regular PA, fewer than 15% of Black women meet the 2008 National Physical Activity Guidelines, the lowest of all adult subgroups [5,6]. Given the connection between physical inactivity and poor asthma outcomes, there is a critical need for interventions to address low rates of PA among Black women with asthma.

Lifestyle PA interventions, such as walking, have been shown to be an effective and sustainable way to engage in regular PA. Research shows that people living with asthma prefer low-intensity activities such as walking, which has a low propensity to cause asthma symptoms [7-9]. Yet, only 2 randomized controlled trials (RCTs) of lifestyle PA (walking) interventions have focused on adults with asthma [10,11]. One occurs in a supervised research or academic setting and the other in the community setting. While both studies found improvements in asthma outcomes (asthma quality of life or asthma control), they were conducted predominantly in White women, and neither assessed the maintenance of effects on asthma control.

Research testing strategies to encourage Black women with asthma to increase their PA is a nascent area of study. While asthma is a known barrier to PA participation due to exercise-induced bronchoconstriction, contextual factors influencing PA engagement among people with asthma have also been identified. This includes previous negative experiences with the disease such as frequent asthma exacerbations; marked limitations in daily living due to asthma; and the effect of extreme weather, pollen, or pollution on asthma [10,12-14]. This is in addition to the many PA barriers urban Black women face, such as social and culturally based preferences for hair and body type, neighborhood characteristics (safety and walkability), family or caregiver responsibilities, and lack of role models or social support [5,15,16].

### A Lifestyle Physical Activity Intervention for Minority Women With Asthma

Culturally tailored lifestyle PA interventions among Black women without asthma are more effective and sustainable than more generalized interventions [17]. However, similar interventions have not been applied to PA interventions in asthma. Using a theory-informed approach, A Lifestyle Physical Activity Intervention for Minority Women With Asthma (ACTION), was developed as one of the first culturally tailored lifestyle PA interventions for Black women with asthma. Pilot work demonstrated the feasibility and acceptability of the intervention and yielded preliminary findings of improvement in asthma outcomes and moderate PA [18]. This study takes the next step to examine the efficacy of a lifestyle walking program designed for Black women with asthma.

### Conceptual Model

**Figure 1:**
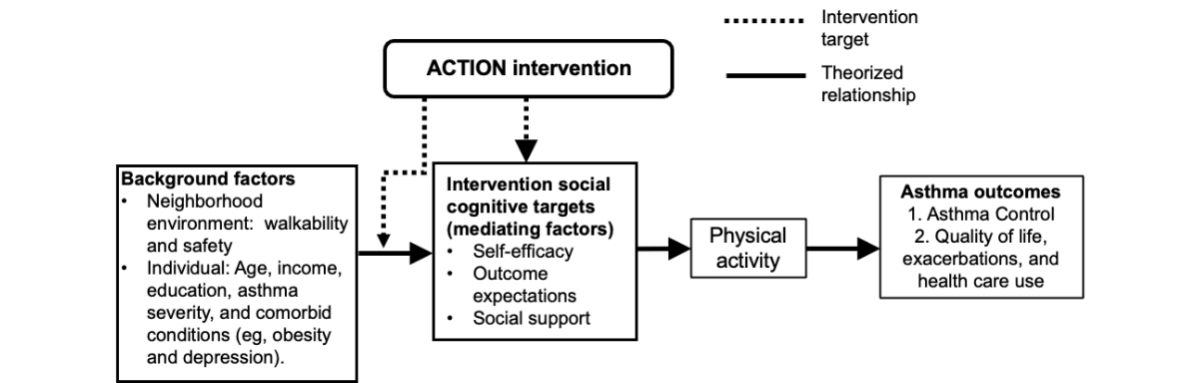
Conceptual framework. ACTION: A Lifestyle Physical Activity Intervention for Minority Women With Asthma.

The conceptual model that guided this project is shown in Figure 1. Within the conceptual framework, explanatory relationships among various determinants of PA or factors that influence behavior, intervention strategies, and subsequent participant outcomes have been examined. Important contextual factors include individual demographics (age, income, and education), disease severity, comorbid conditions, and neighborhood environment. To explain motivation to become more physically active, the intrapersonal characteristics of self-efficacy (belief in one’s ability to perform a behavior), social support, and self-regulation (the process of guiding one’s own thoughts, behaviors, and feelings to reach goals) are used [19].

These intrapersonal characteristics can influence an intervention and thereby influence behavior change. The intervention is targeted to the characteristics of Black women with asthma, such as environmental safety factors, current health, and intrapersonal characteristics. The participant outcomes are those elements that are improved by exposure to the intervention, including PA and health outcomes. The framework specifies that participant outcomes are dynamically related; the greater the tailoring of the intervention to the determinants of the behavior, the greater the likelihood of positive outcomes. Theoretical support for the core elements of the ACTION intervention is derived from social cognitive theory and augmented by self-regulation theory, which stems from social cognitive theory [19,20].

### Objectives and Aims

The proposed study has 3 aims involving a total of 224 sedentary urban Black women with asthma.

- Aim 1A is to determine the efficacy of ACTION intervention on asthma control. We will test the hypothesis that at 24 weeks, Black women with asthma who receive the intervention will have a greater improvement in asthma control (asthma control questionnaire [ACQ]) compared to women in the education control group (efficacy end point).
- Aim 1B is to examine the maintenance effects of the ACTION intervention on asthma control at 48 weeks. We will examine the long-term impact of the intervention on asthma control (eg, control and quality of life). We hypothesize the effects of ACTION on asthma outcomes will be maintained at 48 weeks.
- Aim 2 is to explore the behavioral mediators (eg, self-efficacy, social support, self-regulation, and daily PA levels) and contextual moderators (eg, baseline asthma severity, neighborhood environment, comorbid conditions, and social determinants of health) that contribute to treatment responsiveness. Mediation and moderation analyses will be performed with behavioral mediators and contextual moderators.
- Aim 3 is to assess the reach and implementation potential of ACTION. Reach will be measured by the number of potential participants (based on the recruitment pool) who are randomized into the study. Implementation potential will be measured using a mixed methods approach to identify important explanatory factors underlying the performance of the intervention components.

## Methods

### Project Overview

This study uses a theory-driven intervention (ACTION) to deliver a 24-week lifestyle PA intervention designed for and by urban Black women with asthma. Participants are recruited through 2 urban health care systems that care for a diverse urban Black population. Patients are randomized to one of two groups: (1) ACTION intervention (group sessions, PA self-monitoring, and text-based support for goal setting) or (2) education control (an individual asthma education session and text messages related to asthma education). Participants are followed for an additional 24 weeks after the intervention to assess for the maintenance of intervention effects on asthma health outcomes.

This efficacy study focuses on asthma outcomes, explores behavioral mechanisms of the intervention, and assesses factors that influence its reach and implementation potential.

### Ethical Considerations

This study is conducted in accordance with the Declaration of Helsinki and the International Conference on Harmonization Good Clinical Practice guidelines and approved by the relevant institutional review boards at the University of Chicago (22-0911). The University of Illinois Chicago and the University of Texas at Austin have ceded institutional review board reliance to the University of Chicago institutional review board. This study has been registered at ClinicalTrials.gov (NCT05726487).

### Study Design

**Figure 2:**
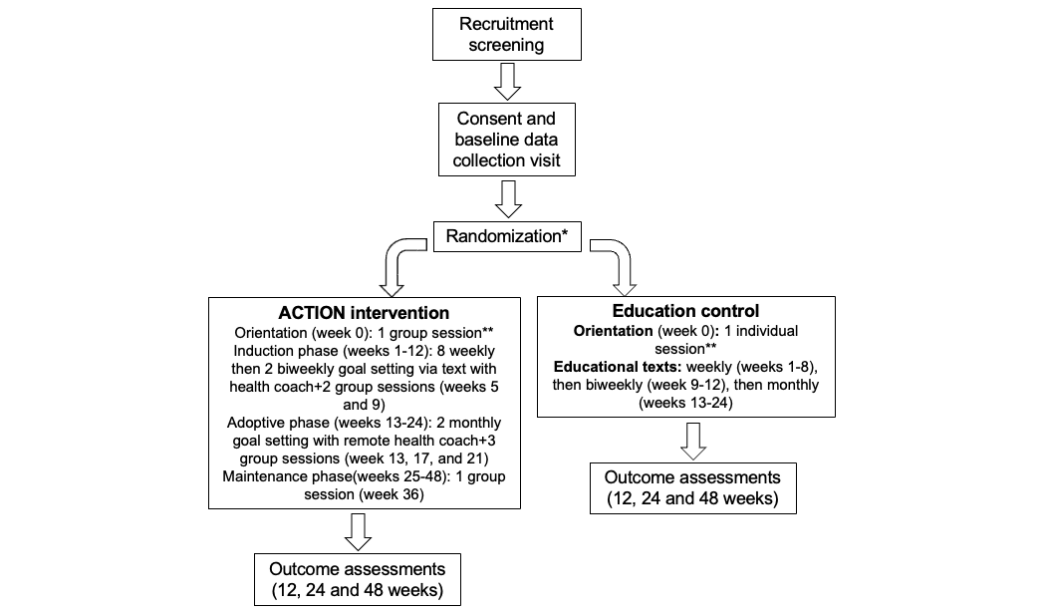
Study design. ACTION: A Lifestyle Physical Activity Intervention for Minority Women With Asthma. *Participant must return accelerometer and have adequate data (10 hours of data for 3 days or more) prior to randomization **Individual/Group sessions are in-person except group session 3 and 5 which are remote.

This is a 2-arm RCT enrolling participants at the University of Chicago and the University of Illinois at Chicago in which 224 Black women with asthma will be randomly assigned to an education control group or receive ACTION intervention for 24 weeks (Figure 2). The remaining 24 weeks is the maintenance phase, and participants in the intervention group will receive 1 booster group session at ~36 weeks. Assessments will occur at 0, 12, 24, and 48 weeks.

The primary efficacy end point is a change from baseline to week 24 asthma control as assessed by asthma control questionnaire-6. We hypothesize that ACTION will be superior to education control for improving asthma control by week 24. The 48-week time point is important for examining behavioral mediators, contextual moderators, and maintenance of intervention effects.

### Participants and Eligibility

#### Recruitment

The primary recruitment strategy uses electronic health records to identify patients who meet the basic inclusion criteria: age≥18 years old, female, Black or African American, International Statistical Classification of Diseases, 10th Revision (ICD-10) diagnosis of asthma, and no ICD-10 diagnosis of chronic obstructive pulmonary disease (Textbox 1). An electronic health record data of patients meeting these criteria is obtained for each respective institution. Patient registries, in-clinic referrals, and flyers are also used as recruitment methods. Emails, SMS text messages, and postcards containing information about the study and a link to the eligibility screening survey are sent to randomly selected batches of potential participants.

**Inclusion and exclusion criteria for the study.**

**Inclusion criteria**

- Self-identify as female and Black or African American
- Age≥18 years
- Physician diagnosed asthma that is suboptimally controlled based on Asthma Control Test (<20) or history in the past year of an asthma exacerbation (a need for systemic corticosteroids or hospital admission or emergency treatment for worsening asthma)
- Willing to enroll and provide written informed consent
- Willing to be randomized to 1 of the 2 study arms
- Has a smartphone or tablet with unlimited texting capabilities and uses text messaging at least daily

**Exclusion criteria**

- Plans to move from the Chicagoland area during the study period
- Unable to ambulate without the use of a wheelchair or scooter
- Currently pregnant, less than 3 months postpartum, or pregnancy anticipated during the study
- Significant medical (eg, unstable heart disease, uncontrolled high blood pressure, active cancer treatment in the past 1 year, and end-stage organ failure) or psychiatric (eg, active bipolar disorder and psychosis) comorbidities
- Diagnosis of chronic obstructive pulmonary disease (emphysema or chronic bronchitis) suggested by patient report of doctor diagnosis or smoking history (≥20 pack year history)
- A contraindication to exercise as indicated by the exercise assessment and screening tool unless written permission by a health care provider
- Participation in another structured physical activity program
- Asthma exacerbation, defined by an urgent care visit for asthma in the last 4 weeks, or the need for an acute course of systemic corticosteroids for asthma in the last 4 weeks
- Family or household member of another participant or staff member
- Inability to speak, read, or understand English
- Investigator discretion for safety or protocol adherence reasons

### Eligibility Screening

Potential participants can self-screen using a REDCap (Research Electronic Data Capture; Vanderbilt University) eligibility screening form. This screener is accessed by scanning a QR code on this study’s flyers and postcards or through a link that may be emailed or texted to potential participants. This survey is also verbally administered by the study staff over the phone.

If potential participants are immediately eligible, they are asked to schedule their baseline visit. Those who do not immediately meet the eligibility criteria or need medical clearance will be contacted by the study staff.

### Baseline Assessment, Randomization, and Blinding

The baseline visit begins with the informed consent process facilitated by study staff. Participants are asked to complete study surveys (patient demographics, asthma demographics, Asthma Control Questionnaire, Asthma-related Quality of Life Questionnaire, Asthma Safety Measures, Adult Asthma Adherence Questionnaire, Patient Health Questionnaire Depression Scale Scored, PROMIS SF v 1.0-Sleep Disturb, SDQ-SA, PWMAQ, Exercise Self-Regulation, Self-efficacy for Walking Scale, Social Support for Walking Scale, Social Support and Exercise Survey, Active Where); baseline spirometry [21] and anthropometrics are obtained by trained study staff. Participants at 1 site are also asked to wear an accelerometer for 7 days.

Eligible participants will be randomized following recruitment and of all baseline measures, using stratified randomization in REDCap at each site after baseline data collection is complete. Stratification will be based on age (18-45 and 46+ years) and current BMI (≤25 and >25 kg/m^2^). Blinding of participants is not feasible for this study. Participant’s health care providers are blinded to group allocation.

### Intervention

#### Overview

ACTION is a behavioral lifestyle intervention that promotes lifestyle PA in accordance with the current PA guidelines in the United States [17]. This intervention is a 24-week group lifestyle PA intervention consisting of 7 group sessions, PA self-monitoring, and text-based support for goal setting. The active period of the intervention is 24 weeks. Trained interventionists and remote health coaches deliver the intervention via a combination of in-person group walking sessions and text-based goal setting. Trained research staff provide a Fitbit for the participant and deliver the text messaging component of the intervention.

#### Group Sessions

##### Session 1

Participants attend an in-person or Health Insurance Portability and Accountability Act (HIPAA)–compliant audio or video platform (eg, Zoom) group session on “Becoming Physically Active with Asthma.” The group session includes a 45-minute asthma education session, based on the American Lung Association’s Asthma Basics course, led by a Black, female asthma educator. Participants in the intervention group are given a manual with educational materials, which include information on increasing PA with asthma. Following the education content, each intervention group participant receives a Fitbit Charge 5 for self-monitoring of steps and heart rate. Specific step-by-step instructions on setup and use of the Fitbit monitor are provided with individual support as needed in the group setting. The study team reviews Specific Measurable Attainable Relevant and Time bound (SMART) goal setting [22], assesses text preferences, and sets individualized step goals based on their baseline PA levels.

##### Sessions 2-7

Women attend a 2-hour, in-person, or video remote group meeting at a convenient time during the induction or adoptive phases (weeks 0-24) and once during the maintenance phase (weeks 25-48). Participants from the individual sites are kept separate and do not attend group sessions together. Group session content is described in Textbox 2.

After 24 weeks of active intervention, participants randomized to ACTION no longer receive remote health coach text messages and attend monthly group sessions. A booster group session at 36 weeks will occur for the intervention group only.

**Content of ACTION (A Lifestyle Physical Activity Intervention for Minority Women With Asthma) group sessions.**

**Session 1 (week 0)**

- Safely exercising with asthma: “Becoming Physically Active with Asthma”
- Receive Fitbit and discuss setting step goals

**Sessions 2-7 (weeks 5,9,13, 17, 21, and 36)**

- Video (10 minutes) on physical activity topics (eg, identifying realistic expectations for PA outcomes with asthma, handling personal and environmental problems that interfere with physical activity, and handling relapses)
- Group discussion and problem-solving (45 minutes)
- Group stretching and walking (20 minutes)

#### PA Tracking as an Intervention Component

Each intervention participant is given a Fitbit Charge 5 that counts steps per day, distance, active minutes (time spent in moderate to vigorous activity), sedentary time, and heart rate. Participants work with health coaches (as above) to set their step goals and monitor their PA on the Fitbit App. Participants are required to download the Fitbit app on their phone or tablet. The Fitbit mobile app and device require the creation of individual digital accounts with Google for all study participants. During the study, participants will use their study-created Google account to interact with the features of the Fitbit mobile app. Upon completion of the program, participants will be able to transfer their Fitbit device to an account using their personal Google login for continued use of the device.

**Figure 3:**
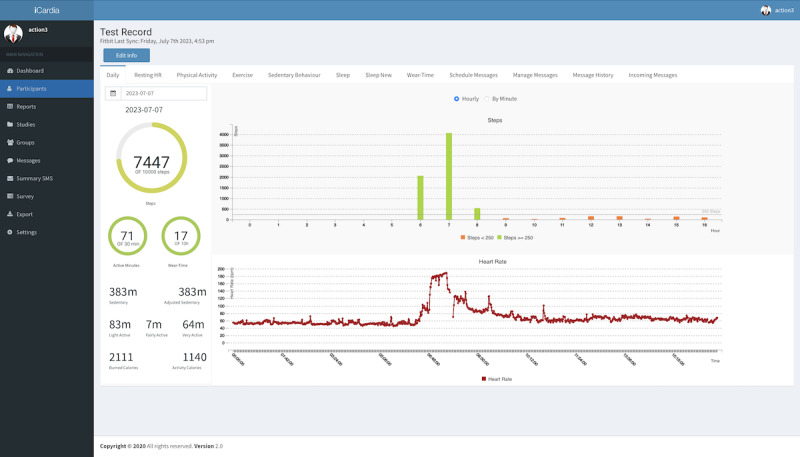
Screenshot of the iCardia platform.

Fitbit data are managed through iCardia. The iCardia platform [23] is used to remotely collect real-time PA data from study participants’ Fitbit app and to send personalized SMS text messages to participants’ cell phones (Figure 3). iCardia (University of Illinois at Chicago) is a secure password-protected system that comprises user-friendly visualization and data exportation tools, allowing key research personnel to view participants’ Fitbit data in the form of graphs every time participants sync their wearable tracker with the Fitbit mobile app. Study participants do not have access to iCardia; they will only interact with the Fitbit mobile app and activity tracker. The SMS text messaging feature is integrated with the iCardia clinical dashboard and uses Twilio’s application programming interface to send messages via the SMS protocol.

#### Remote Health Coach and Individualized Step Goal Setting and Tracking

Study participants will work with the health coach through texts to set a SMART goal [22]. This will begin within 1 week of the completion of session 1. The process includes contingency planning (what if…). Goals will be set once a week for 8 weeks, every 2 weeks for weeks 9-12 sessions, and monthly through 24 weeks. We have set a realistic PA goal for intervention participants of 3000 steps per day of moderate intensity above each participant’s baseline (based on Fitbit-based step counts per day), recognizing that baseline levels of activity will vary across individuals. Thirty minutes of walking at moderate intensity produces an estimated average of 3000 steps in most free-living adults [24]. Health coach texting will be managed through iCardia (described above). Health coaches can monitor Fitbit steps to evaluate goal attainment and send texts to support self-regulation via the text messaging protocol. The Fitbit data collected in iCardia will be used to generate process indicators but not as the primary measure to assess changes in health-enhancing PA (moderate to vigorous intensity physical activity [MVPA]). Health coaches will be sensitive to health status that may impact the ability to escalate steps. Weekly meetings will be conducted with health coaches to ensure texts are following the SMART goal framework and discuss any safety issues or questions.

#### Education Control Group

The education control group will receive (1) 1 group asthma education session similar to session 1 described above and (2) text messages with asthma facts and tips (see Table S1 in Multimedia Appendix 1). The asthma education session consists of a 45-minute video on basic asthma education based on American Lung Association’s asthma basics content followed by a live Q&A with an asthma educator. The video was culturally tailored using feedback from stakeholders and includes a Black female asthma educator and other Black women with asthma. The SMS text messages with asthma facts and tips were also developed using feedback from stakeholders and will be delivered twice weekly (weeks 1-8), biweekly (weeks 9-12), and monthly (weeks 13-24). At the completion of the study, participants in the education control group will receive the study manual, a pedometer, and access to the videos presented in the ACTION intervention group sessions.

### Measures

#### Asthma Measures

Asthma control will be measured using the validated ACQ [25]. Asthma quality of life will be measured using the mini-asthma quality of life measure, a well-validated survey [26]. Asthma exacerbation will be defined as the need for systemic corticosteroids, hospital admission, or emergency treatment for worsening asthma. This will be self-reported, and the time frame will be captured at 3- and 6-month time frames (Table S2 in Multimedia Appendix 2). Health care use is defined as the number of urgent care or emergency room or hospitalization visits. This will be self-reported, and the time frame will be in 3- and 6-month time frames (Table S2 in Multimedia Appendix 2). Spirometry will be performed using standard procedures at baseline, 24, and 48 weeks. Asthma severity will be assessed per the National Heart Lung and Blood Institute National Guidelines for Asthma management based on current asthma treatment [27].

#### PA Measures

Given that the intervention centers on increasing PA levels through walking, it is important to accurately assess the changes in health-enhancing PA among intervention and control participants throughout the study period. Health-enhancing PA is defined as having MVPA and can occur in the domains of discretionary time, travel, home, or work [17]. Our intervention focuses on increasing overall MVPA levels by adding time spent in moderate-intensity PA during discretionary time, through walking. We will measure domain-specific PA through self-reported data for the full sample. We will also measure PA objectively in half of the sample, as this is a more cost-prohibitive assessment instrument.

#### Accelerometry

MVPA (minutes per day) at baseline (before randomization), 12, 24, and 48 weeks will be assessed objectively using accelerometry. During each assessment period, accelerometer data will be collected over 7 consecutive days using the ActiGraph wGT3X-BT activity monitor (ActiGraph, LLC), a small, triaxial piezoelectric accelerometer (4.6 cm×3.3 cm×1.5 cm; 19 g) that will be worn around the waist on an elastic belt. Accelerometry data will be collected using a standardized protocol. ActiGraph monitors have been extensively validated in laboratory and free-living conditions [28]. At the first data collection appointment, study staff will introduce the accelerometer and provide participants with verbal instructions and a package that includes (1) an initialized accelerometer, (2) detailed written wear instructions, (3) tracking log, (4) contact information, and (5) a padded prepaid trackable envelope to return the devices after concluding the 7-day wear period.

In the tracking log, participants will enter the (1) time they put the monitor on in the morning, (2) time removed it in the evening, and (3) time that the monitor was removed and replaced for ≥30 minutes throughout the day (eg, bathing or swimming activities). Raw triaxial data will be sampled at 40 Hz for 7 consecutive days. Once the accelerometer and tracking log are returned by the participant, data will be downloaded by the study staff and prepared for processing and analysis. Data will be processed using ActiLife software (ActiGraph) through a digitally matched filter, reintegrated up to a 60-s epoch, and screened for wear time using the Choi algorithm [29]. Weekly summary accelerometer estimates will be averaged (across days) for all participants with ≥4 valid days of ≥10 hours per day of wear time (minimum wear time to consider the data valid for analysis). If insufficient wear time is detected, the participant will be contacted and asked to rewear the device to provide valid data. Average accelerometer counts (per minute per day) will be calculated using summed daily counts detected over wear periods, and vector magnitude will be calculated as the square root of the sum of the squares obtained from each axis (ie, 3) of data. Time (ie, minutes per day) spent sedentary and in light-, moderate-, and vigorous-intensity physical will be estimated using Freedson cut points (for adults) based on triaxial data [30]. Data checks, including a descriptive analysis of the accelerometer estimates, will be performed quarterly.

#### Self-Reported Discretionary Time and Travel-Based PA

Self-report data adds rich context that accelerometers lack [31]. Our self-report measures will provide information on the domain (eg, recreational and transportation) of PA. These data will be important to quantify the effect of the intervention in increasing leisure-time walking (its main intention), as well as any potential unintended consequence in increasing or decreasing time spent on other types of activities. Participants will self-report PA using the Past Week Modifiable Activity Questionnaire (PWMAQ). The Modifiable Activity Questionnaire is a self-report instrument that assesses leisure PAs over the past 7 days [32,33]. The structure of the PWMAQ is similar to the Modifiable Activity Questionnaire and includes questions that quantify occupational activity (eg, sedentary, low, and high active occupations), active transport, and common sedentary behaviors. The PWMAQ includes information on 38 leisure-time PAs common among diverse adults. The PWMAQ has been shown to be a reliable and valid measure of PA compared to accelerometry and physical fitness [34,35].

#### Additional PA-Related Measures

Self-efficacy for walking, social support for exercise, and self-regulation will also be assessed as they constitute behavioral mediators for attaining increased PA levels, and the physiologic response to increases in PA, respectively. Details on these measures can be found in Table S3 in Multimedia Appendix 3.

#### Contextual Moderators

Contextual moderators will be collected at the in-person baseline data collection visit. The neighborhood environment will be assessed using a validated survey about neighborhood safety, crime, and areas to engage in PA [36]. Social determinants of health, including age, income, education level, marital status, and economic hardship will be assessed using validated questions that the principal investigator has used in prior studies. These have been chosen as they can impact PA levels [37,38]. The comorbid conditions of interest are obesity and depression as they are known to moderate PA levels and asthma outcomes [39,40]. Obesity is defined as a BMI ≥30 kg/m^2^, and depression will be assessed by a validated depression questionnaire, Patient Health Questionnaire Depression Scale-8 [41].

#### Implementation Measures

We propose an explanatory sequential mixed methods design in which quantitative analyses are followed by qualitative analyses to maximize our understanding of the implementation of ACTION [42,43]. The quantitative data will include detailed process metrics and surveys, and qualitative data will include brief interviews with participants. Process metrics will be collected based on the number of participants screened and randomized. Surveys to assess the acceptability of the intervention components will be collected at the end of each group session and at the end of the intervention (after group session 6). Evaluations of the intervention will be conducted using open-ended questions with intervention participants at 24- and 48-week data collection visits. Questions will focus on aspects of the intervention they found helpful, barriers to engagement in each component of the intervention (eg, groups, remote health coach, and Fitbit), and suggestions for improvement. General prompts will be used to orient qualitative data descriptively on the core intervention components. The qualitative portion of the intervention will be conducted by trained and experienced data collectors at the end of the brief quantitative data collection, audio-recorded, and professionally transcribed.

### Data Analysis Plan

Data will be analyzed based on the intent-to-treat principle in this multisite RCT. We will use descriptive statistics to describe the sample by intervention group, and any assumption behind each statistical method will be examined during the analysis. Missing data will be handled by each model’s assumption or by using multiple imputation with a fully conditional specification approach, as appropriate [44]. A 2-sided *P* value less than .05 would be considered statistically significant, and the exact *P* value will be reported. All quantitative analyses will be conducted by using SAS (version 9.4; SAS Institute Inc) and R (version 4.0.2; R Core Team).

The primary research outcome for aim 1A is the differences in ACQ score between baseline and 24 weeks for aim 1A and between baseline and 48 weeks for aim 1B. This will be compared between education control and ACTION intervention groups first using 2 sample *t* test to examine the efficacy and the maintenance effect of the proposed intervention, respectively. Two-tailed statistical tests will be conducted throughout the entire analysis unless specified otherwise. A linear mixed model with a subject-level random intercept then will be built on the same outcome in its original scale having time (12 weeks vs baseline; 24 weeks vs baseline; 48 weeks vs baseline), group (ACTION vs education control), and their interaction as the main factors while adjusting for site, recruitment wave, and any imbalanced confounder [45]. This model will allow us to estimate the efficacy of the intervention through the interaction terms after controlling for confounders and subject dependence. Moreover, any missing data can be handled by this model under the missing at random assumption. The estimated coefficients on the mean scale of the outcome will be reported, along with their SEs, 95% CIs, and *P* values. The similar modeling approach mentioned above using generalized linear mixed models instead will be applied to secondary outcomes (asthma quality of life, health care use, and asthma exacerbations) as secondary analyses.

For aim 2, we will explore the potential mediation effects of multiple variables on the relationship between the intervention and clinical outcomes. That is, a portion of the intervention effect on an outcome may be going through the path via a mediator indirectly. Through mediation analysis, the direct effect, the indirect effect also known as the mediated effect, and their total effect will be measured using the approach by Yu et al [46,47] along with their CIs via bootstrapping. The multiple mediators of interest here include social support, self-regulation, self-efficacy, and PA, while the clinical outcomes of interest are the ACQ score and mini-AQLQ score. To avoid the problem of mediation analysis with cross-sectional data, we define the mediation relationship generally noted as *X*-*M*-*Y*, using *X_BL_* as the exposure at baseline, *M_j_*_,24w_ as the *j*th mediator at 12 weeks (*j*=1,…, 6), and *Y*_k,48w_ as the *k*th outcome at 48 weeks (*k*=1,2) [48]. As a secondary analysis, multilevel mediation analysis using the method by Yu and Li [49] will also be considered. For moderation analysis, we will examine moderators including comorbid conditions (depression and obesity), social determinants of health (age, income, and education level), asthma severity (mild, moderate, and severe), and neighborhood environment (walkability and safety) by separately adding their interaction terms with the treatment group to the models mentioned for aim 1A to estimate their moderating effects on treatment responsiveness. We will focus on several plausible behavioral mechanisms. We anticipate improved PA will lead to improved asthma outcomes (control and quality of life) and a reduction in self-reported exacerbations and emergency health care use.

For aim 3, we will report process metrics, such as recruitment rate, monthly to assess reach overall, within key patient subgroups, and each site. Simple descriptive statistics and 95% CIs will be used to summarize measurement for reach and implementation potential at the end of the study. To identify participant characteristics (eg, age, income, and education level) or site-level factors that may be associated with reach, we will examine those associations using the chi-square test or Fisher exact test for categorical variables and the Kruskall-Wallis test for continuous variables. For analysis of qualitative data, the deidentified interview transcripts will be uploaded to the qualitative data software (eg, NVivo [OSR International] or Dedoose [University of California, Los Angeles]). Qualitative assistants will work with qualitative experts to code the data. All coders will initially read all 112 transcripts to become familiar with the data. Thematic interpretation will focus on individual, social, and structural factors associated with acceptability and reasons for variability in uptake and effectiveness of the intervention components. Ten transcripts will be independently coded by all coders. The codes will be reviewed and discussed collectively to develop a shared understanding of how the codes should be applied to the transcripts. This process of independent coding will be repeated ≥2 times until a consistent agreement is reached. Coded transcripts will be sorted to identify thematic groupings. The thematic groupings will be reviewed to identify emergent themes within each domain of the coding framework and quotes that best represent each domain.

## Results

This project received funding in August 2022 and received institutional review board approval in July 2022. The first year of this project was focused on hiring and training staff, regulatory approvals, setting up data collection processes, and recruitment. We pilot-tested our recruitment and intervention procedures and began recruitment in April 2023. At the time of manuscript submission, 76 participants have been recruited. We anticipate study completion in 2027.

## Discussion

### Principal Findings

As one of the first lifestyle PA interventions in asthma to explore behavioral mechanisms, and the only focusing on a group most in need—Black women, this study is expected to contribute importantly to this very limited body of literature and offer useful insights for PA interventions in asthma. Our intervention focuses on walking as a lifestyle approach in urban Black women with asthma, which has been shown to be the preferred method of PA among Black women and people living with asthma. Walking is affordable, accessible, and sustainable even for Black women residing in urban neighborhoods with high levels of crime or low-resourced neighborhoods. This approach to PA also considers activities done in leisure, household, transportation, and occupational roles. We also use a patient-centered perspective, which has yet to be used in developing PA interventions in adults with asthma. Modifications to the intervention leverage the high levels of technology use in this population while reducing the participant burden associated with in-person sessions [50]. Moreover, from a public health standpoint, interventions using technology-based platforms can provide researchers the ability to reach a large number of people at a relatively low cost, which ultimately leads to a greater public health impact.

### Anticipated Challenges and Limitations

As this study will take place in the Midwest, there is a concern that the weather may have an impact on the ability to engage in walking during the winter and even summer months. The intervention addresses this through providing free or low-cost resources on where to exercise indoors during challenging weather. Furthermore, the study team tailors the text messages to local weather and air quality alerts. For example, during the Canadian wildfires in July 2023 when Chicago was experiencing the poorest air quality in the world, we cautioned participants to monitor the air quality index before going outdoors but also provided alternatives to walking outdoors. During our pilot, the participants requested more flexibility with the in-person sessions and have the option to remote in with a secure video platform, which we incorporated into this study. There may still be challenges in having participants attend all the in-person group sessions, but we will work with the participants to find an optimal time to attend, and if needed, allow for a hybrid group session model with some participants in-person and others virtual. Although not an issue in pilot work, it is possible that differential dropout across study groups may occur. To mitigate this concern, participants in the control arm will receive asthma education session text messages at a similar frequency as the intervention participants. In the pilot study, the asthma education session was valued by participants in the control arm. Nonetheless, should problems arise, coinvestigators and a recruitment or retention core will be engaged for input on changes to improve retention. Finally, the information gained from our implementation measures will be critical for revising the conceptual model and rethinking the choice of intervention components to be tested in future studies and will also inform theory development in implementation science.

### Conclusions

The findings from this study will provide insight into the next steps for addressing PA in Black women with asthma. It will deliver a new approach to PA interventions in chronic lung diseases such as asthma and a better understanding of the underlying behavioral mechanisms among a high-risk underresearched population. This field is in its infancy and showing the short- and long-term efficacy of a culturally tailored lifestyle PA intervention in a vulnerable patient population, as well as identifying the behavioral mechanisms, are important and will be critical. This study will also set a standard for future studies in minority populations with other disease conditions associated with low levels of PA.

## Appendix

Multimedia Appendix 1 Sample asthma education text messages.

Multimedia Appendix 2 Study procedures occurring at each time point.

Multimedia Appendix 3 Outcome measures and variables for A Lifestyle Physical Activity Intervention for Minority Women With Asthma (ACTION).

Multimedia Appendix 4 Peer-review report by the National Institute of Minority Health and Disparities.

## Abbreviations

ACQ: asthma control questionnaire
ACTION: A lifestyle Physical Activity Intervention for Minority Women With Asthma
HIPPA: Health Insurance Portability and Accountability Act
ICD-10: International Statistical Classification of Diseases, 10th Revision
MVPA: moderate-to-vigorous intensity physical activity
PA: physical activity
PWMAQ: Past Week Modifiable Activity Questionnaire
RCT: randomized controlled trial
REDCap: Research Electronic Data Capture
SMART: Specific Measurable Attainable Relevant and Time bound
