# Supplementary material for: Physical Activity Intervention for Urban Black Women With Asthma: Protocol for a Randomized Controlled Efficacy Study
Source: JMIR Res Protoc. 2024 Feb 7;13:e55700. doi: 10.2196/55700 (PMC10882465; doi:10.2196/55700)
Supplement: Multimedia Appendix 1 [file resprot_v13i1e55700_app1.docx]

|  | |
| --- | --- |
| **Medication Adherence** | **Asthma Triggers** |
| If asthma is holding you back from exercising, take control today:  1. Use your rescue inhaler 15-20 minutes before activities 2. Ease into a workout by making sure you warm up and cool down 3. Check pollen & air quality before going outside | Friendly reminder to check the Air Quality Index levels and to avoid outdoor activities on days with poor air quality (AQI > 100) |
| Keys, wallet, phone and rescue inhaler, check! Never leave home without easy access to your inhaler, it’s a lifesaving medication that is just as important as your everyday essentials. | Fun Fact: We spend 1/3 of our home time on average in the bedroom. Bedding attracts allergens and irritants so make sure you wash bedding at least once a week & don’t forget your pillows! |
| Never used a spacer? A spacer helps deliver the right amount of medicine to your lungs and reduces wastage of your hard earned   . If you don’t have one, ask your healthcare provider today! | Are you having wheezing, chest tightness, or trouble breathing? This is a red flag that your asthma is not controlled. Call your healthcare provider to discuss your asthma. |
| Voice hoarse? Your steroid inhaler may irritate your throat. Try rinsing and gargling w/ water after each use and make sure to spit it out after! | Don’t let the fungus among us trigger your asthma. Cold, dark and moist places can harbor mold & cause uncontrolled asthma symptoms. |
| If your asthma is out of control, remember to minimize triggers, use your rescue medication 15-20 minutes before exercise and put your asthma action plan into action! | If you’re sensitive to mold, try these helpful tips: 1. removing moisture by using a dehumidifier or fans to circulate air 2. open windows if possible 3. spray affected areas w white vinegar & let air dry. |
| Using a steroid inhaler? Make sure to gargle & rinse your mouth after each dose to prevent problems. A spacer can help. Contact your healthcare provider for more info. | Don’t forget to clean your inhalers/spacers! Keeping them clean, dry and stored in a cool place to help avoid breathing in dust from your mouthpiece. |
| A healthy lifestyle includes routine vaccination. Living with asthma makes getting vaccinated even more essential to living your best. | When you’re using your rescue inhaler more than three times a week is a red flag. Call your healthcare provider to discuss your asthma. |
