# Supplementary material for: Physical Activity Intervention for Urban Black Women With Asthma: Protocol for a Randomized Controlled Efficacy Study
Source: JMIR Res Protoc. 2024 Feb 7;13:e55700. doi: 10.2196/55700 (PMC10882465; doi:10.2196/55700)
Supplement: Multimedia Appendix 2 [file resprot_v13i1e55700_app2.docx]

|  | | | | | |
| --- | --- | --- | --- | --- | --- |
|  | Baseline | 12 weeks | 24 weeks | 48 weeks | Group Sessions |
| Patient demographics | X |  |  |  |  |
| Social determinants of health measures (e.g., income, neighborhood environment, tobacco smoke exposure) | X |  | X | X |  |
| Co-morbid conditions (e.g., depression, obesity) | X |  | X | X |  |
| Asthma surveys | X | X | X | X |  |
| Spirometry | X |  | X | X |  |
| Exercise surveys (self-efficacy, social support, self-regulation) | X | X | X | X |  |
| Physical Activity measures (Accelerometry and self-reported) | X | X | X | X |  |
| Implementation measures (Intervention Acceptability) |  |  | X | X | X |
| Participant reimbursement | X | X | X | X |  |
| Travel reimbursement | X |  | X | X | X* |

*in-person only
