# Supplementary material for: Physical Activity Intervention for Urban Black Women With Asthma: Protocol for a Randomized Controlled Efficacy Study
Source: JMIR Res Protoc. 2024 Feb 7;13:e55700. doi: 10.2196/55700 (PMC10882465; doi:10.2196/55700)
Supplement: Multimedia Appendix 3 [file resprot_v13i1e55700_app3.docx]

| Variables and instruments | Description |
| --- | --- |
| Patient demographics and social determinants of health | Age, address, race or ethnicity, education, employment, marital status, family income, children, economic hardship, neighborhood environment (Physical Activity Neighborhood Environment Survey, perceived neighborhood safety, and violence), insurance status, tobacco exposure, and comorbid conditions (PHQ-8 for depression) |
| Vital signs and anthropometrics | Blood pressure, heart rate, height (stadiometer), and weight (digital scale) will be performed by trained data collectors. BMI will be calculated as weight (kg)/height (m)^2^ |
| Descriptive asthma measures:   - Asthma severity - Spirometry | - Assessed per the NHLBI National Guidelines for Asthma Management based on current asthma treatment (mild, moderate, severe) - Spirometry using a standardized protocol will be performed |
| **Aim 1A/1B—asthma outcomes** | |
| Asthma control questionnaire (ACQ-6; 1) | A 6-item validated questionnaire will be used to classify asthma control (minimal clinically important difference-MCID=0.5 change) |
| Mini-asthma quality of life questionnaire (mini-AQLQ; 2) | A 32-item validated asthma-specific instrument will be used to query about quality of life (minimal clinically important difference-MCID=0.5 change) |
| Asthma exacerbations (2) | Defined as a need for systemic corticosteroids or hospital admission or emergency treatment for worsening asthma (self-report) |
| Health care use (2) | Number of self-reported urgent care, emergency room visits, and hospitalization for asthma |
| **Aim 2—behavioral mediators** | |
| Self-efficacy for walking scale | Assesses participants’ beliefs in their physical capability to successfully complete incremental 5-minute intervals (5 to 40 minutes) at a moderately fast pace |
| Social support for exercise survey | Assess the perceived level of support from family and friend regarding making health-behavior changes (exercise) |
| Self-regulation:   - Exercise Self-regulation questionnaire - Step goals achieved | - Assesses motivational regulations toward physical activity, contains the subscales external regulation (α=.73), and identifies regulation (α=.88) and intrinsic motivation (α=.88) - Number of days per week participation achieve their step goals as measured by Fitbit |
| PA levels   - Actigraph GT3XP-BTLE waist-based Accelerometer - Past Week Modifiable Activity Questionnaire | - The ratings of activity (number of steps per day and the amount of time spent in sedentary, low, moderate, and vigorous physical activity (100 steps per minute) will be collected for a minimum of 10 hours per day for 3 days - Assessing leisure physical activities over the past 7 days. Output includes number of hours (or metabolic equivalent-MET hours) per week of physical activity |
| **Aim 3: Implementation potential** | |
| Process metrics | Examine reach across sites |
| Surveys | Likert scale to assess acceptability of ACTION intervention components including technology components |
| Semistructured interviews | Understand acceptability and reasons for variability in uptake and efficacy of ACTION |
| Presentation | Seek input from asthma community advisory board for future intervention scale up |
