# Supplementary material for: Physical Activity Intervention for Urban Black Women With Asthma: Protocol for a Randomized Controlled Efficacy Study
Source: JMIR Res Protoc. 2024 Feb 7;13:e55700. doi: 10.2196/55700 (PMC10882465; doi:10.2196/55700)
Supplement: Multimedia Appendix 4 [file resprot_v13i1e55700_app4.pdf]

SUMMARY STATEMENT

(Privileged Communication)

PROGRAM CONTACT:
Nathaniel Stinson
301-594-8704
stinsonn@mail.nih.gov

Release Date: **10/26/2021**
Revised Date:

Application Number: **1 R01 MD017277-01**

Principal Investigator

NYENHUIS, SHARMILEE MARIA

Applicant Organization: UNIVERSITY OF ILLINOIS AT CHICAGO

Review Group: **BMHO**
Biobehavioral Medicine and Health Outcomes Study Section

Meeting Date: **10/04/2021**
Council: **JAN 2022**
Requested Start: **04/01/2022**

RFA/PA: **PA20-183**
PCC: **CPS12JA**
Dual IC(s): **HL, AI**

Project Title: **ACTION, A lifestyle physiCal acTivity Intervention for MinOrity womeN with asthma: From Efficacy to Implementation**
SRG Action: **Impact Score:27 Percentile:13**
Next Steps: **Visit https://grants.nih.gov/grants/next_steps.htm**
Human Subjects: 30-Human subjects involved - Certified, no SRG concerns
Animal Subjects: 10-No live vertebrate animals involved for competing appl.
Gender: 2A-Only women, scientifically acceptable
Minority: 2A-Only minorities, scientifically acceptable
Age: 1U-Children, Adults, Older Adults, scientifically unacceptable

Project Year	Direct Costs Requested	Estimated Total Cost
1	498,018	806,175
2	499,999	809,381
3	499,999	809,381
4	495,220	801,645
5	438,930	710,525
TOTAL	2,432,166	3,937,107

ADMINISTRATIVE BUDGET NOTE: The budget shown is the requested budget and has not been adjusted to reflect any recommendations made by reviewers. If an award is planned, the costs will be calculated by Institute grants management staff based on the recommendations outlined below in the COMMITTEE BUDGET RECOMMENDATIONS section.
NEW INVESTIGATOR

1R01MD017277-01 Nyenhuis, Sharmilee

INCLUSION ACROSS THE LIFESPAN PLAN UNACCEPTABLE
NEW INVESTIGATOR

RESUME AND SUMMARY OF DISCUSSION: This application proposes a randomized clinical trial to test the efficacy and implementation potential of a lifestyle intervention to increase physical activity (PA) and improve asthma health outcomes in African-American (AA) women. During discussion the panel agreed that examining the mechanistic relationship between PA and asthma in AA women is high, with potential to advance clinical treatment by furthering the mechanistic understanding of PA in minority populations with asthma. Reviewers noted this experienced new investigator assembled a strong interdisciplinary investigative team with a history of collaboration and together developed an innovative multi-faceted affordable lifestyle intervention for AA women with asthma incorporating remote health coaching. Additional strengths include strong pilot data with good indices of feasibility and acceptability, an appropriate theoretical framework, well-articulated intervention and control groups, and a rigorous design which includes self-report and objective outcome measures, a sound data analytic plan and a comprehensive intervention fidelity assessment. The panel identified addressable weaknesses such as lack of clarity on how health coaches will be monitored, whether text messages are standardized, whether seasonality is incorporated into the analytic plan, and how potential contamination between treatment and control groups will be managed. Overall, the panel agreed the application's strengths outweighed its weaknesses and will have a moderately high impact on increasing physical activity and improving health outcomes for African-American women with asthma.

DESCRIPTION (provided by applicant): Physical inactivity is associated with poor asthma control and quality of life, and greater health care utilization. Rates of physical inactivity, asthma, and asthma mortality among African American (AA) women are higher than those of their White counterparts. Our formative work identified barriers to PA among African American women with asthma including a lack of social support, self-efficacy, unsafe neighborhood and fear related to experiences with life-threatening asthma exacerbations. Given the unique barriers to PA and high rates of physical inactivity that are associated with poor asthma outcomes in African American women, there is an urgent need to optimize PA interventions for this population. The proposed study uses our theory-driven intervention (ACTION: A lifestyle physiCal acTivity Intervention for minOrity womeN with asthma) to deliver a 24-week lifestyle physical activity intervention designed for and by urban AA women with asthma. Participants will be recruited through two urban health care systems that care for a diverse urban AA population. Patients will be randomized to one of two groups: 1) ACTION intervention (group sessions, physical activity self-monitoring and text-based support for goal-setting), or 2) education control (an individual asthma education session and text messages related to asthma education). Participants will be followed for an additional 24-weeks after the intervention to assess for the maintenance of intervention effects on asthma health outcomes. We are proposing an efficacy study that focuses on asthma outcomes (Aim 1A/B), explores behavioral mechanisms of the intervention (Aim 2) and assesses factors that influence its reach and implementation potential (Aim 3). This trial will provide the first ever evidence of the efficacy of a lifestyle physical activity intervention among urban AA women with asthma, a population that is understudied yet plagued by low levels of PA and poor health outcomes. Our study has high potential to advance clinical treatment of asthma, and further the mechanistic understanding of physical activity interventions in minority populations living in low-resourced urban environments.

PUBLIC HEALTH RELEVANCE: Physical inactivity is associated with worse asthma outcomes, and African American women are disproportionately impacted by both physical inactivity and asthma. Research aimed at addressing the unique asthma-related and culture-specific barriers to physical activity in this vulnerable population of women is needed. The proposed study will test the efficacy and

implementation potential of ACTION (A lifestyle physiCal acTivity Intervention for minOrity womeN with asthma) to increase physical activity and asthma health outcomes to reduce the burden of asthma in African American women.

CRITIQUE 1

Significance: 1
Investigator(s): 2
Innovation: 1
Approach: 3
Environment: 1

Overall Impact: This RCT will test the efficacy and implementation potential of ACTION (A lifestyle physical acTivity Intervention for minOrity womeN with asthma) to increase physical activity and asthma health outcomes to reduce the burden of asthma in 224 African American women. The main hypothesis is that the 24-week intervention (ACTION) will lead to a significant improvement in asthma control that will be maintained at 48-weeks with a less intensive intervention. Aims 1 and 3 are independent and complementary. Importantly Aim3, will provide insight regarding implementation of the intervention, which can have a great public health impact. The research team is strong and well-rounded, the environment is well-suited for such a trial, and the approach is overall sound. There are some weaknesses/missed opportunities mostly related to Aim 2 that could be easily addressed. In summary, the overall impact of this application is strong.

1. Significance:

Strengths

- This RCT is well-aligned with the K award goals and seems to be a natural continuation of the K work
- Preliminary data regarding asthma control and some mediators is strong
- A patient-centric intervention specifically targeting AA women is long due
- Data and methods derived from this RCT could be applied to other outcomes affected by sedentarism, such as cardiometabolic risk and the like

Weaknesses

- None noted

2. Investigator(s):

Strengths

- The PI piloted the exercise program during her K award
- Dr. Sharp is well-experienced in randomized behavioral interventions
- Dr. Kitsiou is expert in mobile health applications and developed iCardia. This is a unique expertise
- Dr. Press has significant experience in implementing self-care strategies in minority population
- Investigators at other sites are well-suited to conduct the trial

Weaknesses

- This is an observation, not a real weakness: PI's total effort for this project is 28% and not 35% as listed.

3. Innovation:

Strengths

- A multi-faceted affordable physical activity intervention for a minority group at risk is novel
- Great use of mobile health and remote health coaching. This a thoughtful approach to reduce participants' burden and promote social distancing in these times
- iCardia platform is innovative and might be able to develop other uses during the duration of this trial

Weaknesses

- None noted

4. Approach:

Strengths

- Well thought-out trial deployment
- Sound sample size calculation and attrition estimation. Hence, the proposed sample size is feasible
- Strong Aim 1 with high possibilities of panning out
- Strong and needed Aim 3, very important from a public health standpoint

Weaknesses

- Aim 2 involves several explorations and hence, is subject to potential pitfalls as described below
- Interventionists and remote health coaches can add variability to the intervention. Will a remediation plan be instituted if needed? These factors should also be considered as moderators in the statistical plan
- Along the same lines, can text messages be standardized?
- Will seasons be incorporated into the analyses and/or randomization schedule?
- Minor: I know this is not a sleep-focused grant but it would be important to add one questionnaire about OSA and one about sleep quality as both can mediate exercise, can be affected by asthma, and OSA is not an exclusion criteria. In fact, some participants may be treated with CPAP. This could be an exploratory aim hypothesizing that sleep quality would improve in the intervention group. Easy to solve missed opportunity
- I understand the constraints of longer trials, but it would be important to explore sustainability of findings by continuing tracking steps 6 months after the end of the trial for example, and correlate these findings with asthma control tools

5. Environment:

Strengths

- UIC has a strong system to foster tis research. This includes the Center for Clinical and Translational Science, the Bioinformatics core, mHealth lab, and a vast clinical population
- UC also has a vast clinical population to support this research
- Letters of support clearly described the facilities and resources available at each institution

Weaknesses

- None noted

Study Timeline:

Strengths

- Appropriate timeline

Weaknesses

- None

Protections for Human Subjects:

Acceptable Risks and/or Adequate Protections

- N/A

Data and Safety Monitoring Plan (Applicable for Clinical Trials Only):

Acceptable

- NIH may set up an independent DSMB for this clinical trial

Inclusion Plans:

- Sex/Gender: Distribution justified scientifically
- Race/Ethnicity: Distribution justified scientifically
- Inclusion/Exclusion Based on Age: Distribution justified scientifically

Vertebrate Animals:

Not Applicable (No Vertebrate Animals)

Biohazards:

Not Applicable (No Biohazards)

Resource Sharing Plans:

Acceptable

- I suggest including the intervention manual in this section. It would be important to share this with other teams interested in this type of research

Budget and Period of Support:

Recommend as Requested

CRITIQUE 2

Significance: 3
Investigator(s): 4
Innovation: 3
Approach: 3
Environment: 2

Overall Impact: Rates of physical inactivity, asthma, and asthma mortality among African American (AA) women are higher than those of their White counterparts The investigators propose a 24-week theory- driven lifestyle physical activity intervention designed for and by urban AA women with asthma. The proposed study has the potential to have a significant scientific impact by advancing clinical treatment of asthma, and furthering the behavioral mechanistic understanding of PA interventions in minority populations living with asthma. The intervention has a strong theoretical framework and is based on preliminary data from the same investigatory team. The investigatory team is heavy with physicians, with a strong biomedical perspective. The study design could be strengthened by the addition of an investigator with experience in women's studies- this would ensure consideration of the norms, values, and the meanings of and the barriers that societies impose on women and their health promotion needs. There are also questions about the potential for contamination of the Experimental and Control groups and how this can be avoided.

1. Significance:

Strengths

- Investigatory team has preliminary data from prior studies that provide a solid premise for conducting this efficacy trial of a PA intervention targeting African American women with asthma
- Intervention is based upon social cognitive theory and augmented by self-regulation theory.

Weaknesses

- Since walking is the principal form of physical activity, have seasonal effects been considered in the design of the study? Chicago winters are cold!

2. Investigator(s):

Strengths

- PI is a new investigator who recently finished a K award, which was the basis for the proposed study
- Investigatory team members share a track-record of collaboration, as evidenced by many funded grant proposals and publications. This is an intradisciplinary team composed of experts in pulmonary medicine, biomedical and health informatics, epidemiology, biostatistics and behavioral science.

Weaknesses

- Three of the investigators (including the PI) are physicians, implying that this proposal likely comes from a strong biomedical perspective. Given that the intervention will be targeting women, I would argue for some epistemological diversity. It would be helpful to have someone with a background in women's studies to put the experiences and situations of women

attempting to exercise within historical, gender, and social contexts. This will inform the intervention to include a consideration of the norms, values, and the meanings of and the barriers that societies impose on women and their health promotion needs.

3. Innovation:

Strengths

- Intervention includes text-based goal-setting with a remote health coach to enhance the PA intervention

Weaknesses

- None noted

4. Approach:

Strengths

- The investigators are proposing an efficacy study that focuses on asthma outcomes, explores behavioral mechanisms of the intervention, and assesses factors that influence its reach and implementation potential.
- Reach of the proposed intervention will be measured by the number of potential participants (based on the recruitment pool) who are randomized into the study. Implementation potential will be measured using a mixed methods approach to identify important explanatory factors underlying the performance of the intervention components.
- Physical activity ill be tracked by accelerometry
- Team plans to recruit a total of 224 subjects- 112 participants in each group. This will assume an attrition rate of 25% from the pilot study.

Weaknesses

- How will you keep individual members of the Experimental and Control groups from contaminating each other?

5. Environment:

Strengths

- Strong environment with ample resources to carry out the proposed study

Weaknesses

- None

Study Timeline:

Strengths

- Seems appropriate

Weaknesses

- None

Protections for Human Subjects:

Acceptable Risks and/or Adequate Protections

- Adequate

Data and Safety Monitoring Plan (Applicable for Clinical Trials Only):
Acceptable

- Acceptable

Inclusion Plans:

- Sex/Gender: Distribution justified scientifically
- Race/Ethnicity: Distribution justified scientifically
- Inclusion/Exclusion Based on Age: Distribution justified scientifically
- Adequate

Vertebrate Animals:
Not Applicable (No Vertebrate Animals)

- N/A

Biohazards:
Not Applicable (No Biohazards)

- N/A

Resource Sharing Plans:
Acceptable

- Adequate

Budget and Period of Support:
Recommended budget modifications or possible overlap identified:

- Adequate

CRITIQUE 3

Significance: 4
Investigator(s): 1
Innovation: 3
Approach: 3
Environment: 3

Overall Impact: Strength of this application in include the focus on a group – African American women – who disproportionately experience negative effects of asthma. PA interventions have been shown to improve asthma control. A lifestyle PA approach is innovative in this particular context, and has been

shown to increase PA in other contexts. The focus on implementation outcomes will help to propel this work forward after the completion of this study. The research team is excellent. The PI has conducted a pilot study of the proposed intervention, and has refined and expanded the intervention based on participant feedback. Investigators include many design features that support rigor of the science. Overall this was a strong application, but I did have some concerns about the study. Most significant concerns are outlined here. First, investigators provide few details on how the intervention was tailored to African American women. Provision of this information would help to justify having an intervention specifically targeted toward this demographic group. Second, there was no theory or hypotheses presented about why increased PA would lead to better asthma control. Third, investigators should use the PHQ-9 rather than the PHQ-8 unless there is a very strong rationale. From a human subjects perspective, it is better to ask about suicide -- as that gives researchers an opportunity to intervene and provide necessary referrals or resources. Fourth, the study should be powered to detect a minimally clinically important differences that is specific to the outcome of interest (asthma control), not general. Power analyses also should not be based on effect sizes from pilot data which likely have a wide confidence interval around them. Finally, there was no institutional information included about the University of Chicago site. Overall, I thought this study has the potential to have a substantive impact if these concerns were adequately addressed.

1. Significance:

Strengths

- African American women have high rates of asthma and low rates of physical activity. People with asthma experience unique barriers to physical activity including fear related to experiences with asthma exacerbations. Previous research has shown that PA interventions are safe and improve asthma-related outcomes. Thus, there is a rationale for using a PA intervention to increase PA an improve asthma control in African American women.
- Investigators are planning to examine efficacy during the active intervention period (24 weeks) as well as maintenance of gains 24 weeks later.
- Investigators plan to investigate increased PA as a mediator of the primary outcome of asthma control.
- Investigators plan to assess implementation outcomes in order to plan for a future scale-up.

Weaknesses

- Investigators do not provide any details on how this intervention was tailored specifically for African American women, other than the fact that the interventionists will be African American women. Providing this information would strengthen the justification for having an intervention specifically targeted toward this demographic group.
- No theory or hypotheses are presented about why increased PA may lead to better behavioral control of asthma.
- Investigators do not provide any hypotheses for the potential moderators that they plan to assess.
- In Aim 3, how will investigators determine that reach and acceptability are adequate?

2. Investigator(s):

Strengths

- Collectively, the team has expertise in multi-site clinical trials, biostatistictics, behavioral interventions for asthma, interventions targeted toward African American women, assessment of PA and behavioral mechanisms, lifestyle PA interventions, mHealth, implementation science, and analysis of qualitative data.
- Dr. Nyenhuis' background in implementation science is a particular strength. They have also conducted the preliminary work (as part of a K01 award) that provides important preliminary data for this proposal.
- The team has a history of working together.

Weaknesses

- None noted.

3. Innovation:

Strengths

- Most previous research on PA interventions for adults with asthma has not involved a lifestyle PA approach.
- Researchers will examine maintenance effects for the lifestyle PA intervention.
- Researchers have tailored the intervention for a high-risk group: African American women.

Weaknesses

- Innovation would be strengthened by more details re: justification for tailoring an intervention specifically for this demographic group.

4. Approach:

Strengths

- Investigators have conducted a pilot study (n=53) of a multicomponent culturally-tailored lifestyle PA intervention for African American women with asthma. This study showed that women in the intervention (vs. control group) significantly increased MVPA. Indices of feasibility and acceptability were good, and feedback from participants was used to refine the intervention. This provides a good basis for the next step in this line of research.
- Investigators provide justification for the length of the intervention phase.
- Interventions and control group are well-described.
- Rigorous aspects of the design include: data collectors who are blinded to treatment group assignment, use of self-report and objective (spirometry, accelerometry) measures of outcome, use of an intent-to-treat approach to data analysis, and a comprehensive approach to intervention fidelity assessment.

Weaknesses

- Investigators might provide a smartphone or tablet to women who don't have one so that they may participate in the study.
- Investigators should use the PHQ-9 rather than the PHQ-8 unless there is a very strong rationale. From a human subjects perspective, it is better to ask about suicide -- as that gives researchers an opportunity to intervene and provide necessary referrals or resources.

- The study should be powered to detect a minimally clinically important differences that is specific to the outcome of interest (asthma control), not general. Power analyses also should not be based on effect sizes from pilot data which likely have a wide confidence interval around them.
- The qualifications or background of the health coaches who engage in goals-based text messaging is unclear. Will these be nurses?
- There is minimal discussion of the theoretical rationale for the various intervention components (e.g., group meeting format, group meeting content, text messaging, provision of Fitbit). Why is this the right combination of intervention components?

5. Environment:

Strengths

- University of Illinois has excellent academic and clinical resources, including strong support for research.
- Investigators have a back-up plan if they are not able to recruit the required numbers from the initial sites.

Weaknesses

- There is no information provided about the University of Chicago site. This site will be responsible for recruiting participants.
- It is unclear whether there is an adequate pool of potentially eligible people from which to recruit participants. Investigators need to recruit 224 women from 8436 African American women in the right age range. If the rate of asthma is 11%, then n= 928 are expected to have asthma. It is unclear how many will also be physically inactive, not pregnant, have sub-optimally controlled asthma, and no medical contraindications. Of those who meet these inclusion criteria, how many will agree to participate?

Study Timeline:

Strengths

- Timeline is likely reasonable, but requires more detail to adequately evaluate.

Weaknesses

- Timeline should be more detailed, with start-up and recruitment milestones, divided into quarters (rather than years).

Protections for Human Subjects:

Acceptable Risks and/or Adequate Protections

- Investigators detail possible risks due to physical activity, with a comprehensive plan for prevention and management.

Data and Safety Monitoring Plan (Applicable for Clinical Trials Only):

Acceptable

- Plan to use an independent safety monitor who will review all SAEs, dropouts, and AEs.

- Given the possibility of SAEs (which may or may not be related to study participation) in this medically ill population, and the size of the trial, investigators and the funding agency may decide that a full DSMB is warranted.

Inclusion Plans:

- Sex/Gender: Distribution justified scientifically
- Race/Ethnicity: Distribution justified scientifically
- Inclusion/Exclusion Based on Age: Distribution not justified scientifically
- Investigators plan to include women aged 18-65. I don't see why they couldn't include older adults provided they meet inclusion criteria. If an older adult has certain physical restrictions (as mentioned in the justification), they would likely not meet inclusion criteria.
- Investigators will enroll African American women only.

Vertebrate Animals:

Not Applicable (No Vertebrate Animals)

Biohazards:

Not Applicable (No Biohazards)

Resource Sharing Plans:

Acceptable

Budget and Period of Support:

Recommend as Requested

THE FOLLOWING SECTIONS WERE PREPARED BY THE SCIENTIFIC REVIEW OFFICER TO SUMMARIZE THE OUTCOME OF DISCUSSIONS OF THE REVIEW COMMITTEE, OR REVIEWERS' WRITTEN CRITIQUES, ON THE FOLLOWING ISSUES:

PROTECTION OF HUMAN SUBJECTS: ACCEPTABLE

INCLUSION OF WOMEN PLAN: ACCEPTABLE

INCLUSION OF MINORITIES PLAN: ACCEPTABLE

INCLUSION ACROSS THE LIFESPAN PLAN: UNACCEPTABLE
The panel agreed that there was insufficient scientific justification for excluding women over the age of 65.

COMMITTEE BUDGET RECOMMENDATIONS: The budget was recommended as requested.

Footnotes for 1 R01 MD017277-01; PI Name: Nyenhuis, Sharmilee Maria

NIH has modified its policy regarding the receipt of resubmissions (amended applications).See Guide Notice NOT-OD-18-197 at https://grants.nih.gov/grants/guide/notice-files/NOT-OD-18-197.html. The impact/priority score is calculated after discussion of an application by averaging the overall scores (1-9) given by all voting reviewers on the committee and multiplying by 10. The criterion scores are submitted prior to the meeting by the individual reviewers assigned to an application, and are not discussed specifically at the review meeting or calculated into the overall impact score. Some applications also receive a percentile ranking. For details on the review process, see http://grants.nih.gov/grants/peer_review_process.htm#scoring.

MEETING ROSTER

Biobehavioral Medicine and Health Outcomes Study Section
Risk, Prevention and Health Behavior Integrated Review Group
CENTER FOR SCIENTIFIC REVIEW
BMHO
10/04/2021 - 10/05/2021

Notice of NIH Policy to All Applicants: Meeting rosters are provided for information purposes only. Applicant investigators and institutional officials must not communicate directly with study section members about an application before or after the review. Failure to observe this policy will create a serious breach of integrity in the peer review process, and may lead to actions outlined in NOT-OD-14-073 at https://grants.nih.gov/grants/guide/notice-files/NOT-OD-14-073.html, NOT-OD-15-106 at https://grants.nih.gov/grants/guide/notice-files/NOT-OD-15-106.html, and NOT-OD-18-115 at https://grants.nih.gov/grants/guide/notice-files/NOT-OD-18-115.html, including removal of the application from immediate review.

CHAIRPERSON(S)

BRUEHL, STEPHEN, PHD
PROFESSOR
DEPARTMENT OF ANESTHESIOLOGY
SCHOOL OF MEDICINE
VANDERBILT UNIVERSITY MEDICAL CENTER
NASHVILLE, TN 37212

MEMBERS

COLLOCA, LUANA, PHD *
PROFESSOR
DEPARTMENT OF PAIN TRANSLATIONAL SYMPTOM SCIENCE
SCHOOL OF NURSING
UNIVERSITY OF MARYLAND
BALTIMORE, MD 21201

D'ALONZO, KAREN T, PHD *
ASSOCIATE PROFESSOR
DIVISION OF NURSING SCIENCE
SCHOOL OF NURSING
RUTGERS BIOMEDICAL AND HEALTH SCIENCES
RUTGERS, THE STATE UNIVERSITY OF NEW JERSEY
NEWARK, NJ 07102

FINAN, PATRICK, PHD *
ASSOCIATE PROFESSOR
PSYCHIATRY AND BEHAVIORAL SCIENCES
JOHNS HOPKINS UNIVERSITY MEDICAL CENTER
BALTIMORE, MD 21224

FORTIER, MICHELLE, PHD
ASSOCIATE PROFESSOR
SUE AND BILL GROSS SCHOOL OF NURSING
UNIVERSITY OF CALIFORNIA, IRVINE
ORANGE, CA 92868

GALLO, LINDA C, PHD
PROFESSOR
DEPARTMENT OF PSYCHOLOGY
SAN DIEGO STATE UNIVERSITY
SAN DIEGO, CA 92123

GOLDMAN, MYLA D, MD *
PROFESSOR
DEPARTMENT OF NEUROLOGY
SCHOOL OF MEDICINE
VIRGINIA COMMONWEALTH UNIVERSITY
RICHMOND, VA 23298

GONZALEZ, BRIAN D, PHD
ASSOCIATE MEMBER
MOFFITT CANCER CENTER
TAMPA, FL 33647

HARPER, FELICITY, PHD
PROFESSOR
DEPARTMENT OF ONCOLOGY
KARMANOS CANCER INSTITUTE
WAYNE STATE UNIVERSITY
DETROIT, MI 48201

HASSETT, AFTON L, PSYD
ASSOCIATE PROFESSOR
DEPARTMENT OF ANESTHESIOLOGY
CHRONIC PAIN AND FATIGUE RESEARCH CENTER
UNIVERSITY OF MICHIGAN MEDICAL SCHOOL
ANN ARBOR, MI 48106

JOHNS, TANYA, MD *
ASSOCIATE PROFESSOR
DEPARTMENT OF MEDICINE
ALBERT EINSTEIN COLLEGE OF MEDICINE
BRONX, NY 10461

KOLTYN, KELLI F, PHD *
PROFESSOR
DEPARTMENT OF KINESIOLOGY
SCHOOL OF EDUCATION
UNIVERSITY OF WISCONSIN - MADISON
MADISON, WI 53706

KRONISH, IAN M, MD
ASSOCIATE PROFESSOR
DEPARTMENT OF MEDICINE
COLUMBIA UNIVERSITY MEDICAL CENTER
NEW YORK, NY 10032

MCGEARY, DONALD D, PHD *
VICE CHAIR FOR RESEARCH AND ASSOCIATE PROFESSOR
DEPARTMENT OF REHABILITATION MEDICINE
SCHOOL OF MEDICINE
UNIVERSITY OF TEXAS HEALTH SCIENCE CENTER
SAN ANTONIO, TX 78229

MEYER, JACOB D, PHD *
ASSISTANT PROFESSOR
DEPARTMENT OF KINESIOLOGY
IOWA STATE UNIVERSITY
AMES, IA 50011

NAPADOW, VITALY, PHD
ASSOCIATE PROFESSOR
MARTINOS CENTER FOR BIOMEDICAL IMAGING
MASSACHUSETTS GENERAL HOSPITAL
HARVARD MEDICAL SCHOOL
CHARLESTOWN, MA 02129

PAGOTO, SHERRY L, PHD *
PROFESSOR
DEPARTMENT OF ALLIED HEALTH SCIENCES
UCONN CENTER FOR MHEALTH AND SOCIAL MEDIA
UNIVERSITY OF CONNECTICUT
STORRS, CT 06269

PERALES PUCHALT, JAIME, PHD *
ASSISTANT PROFESSOR
DEPARTMENT OF NEUROLOGY
UNIVERSITY OF KANSAS
FAIRWAY, KS 66205

PYATAK, ELIZABETH F, PHD
ASSOCIATE PROFESSOR
DEPARTMENT OF OCCUPATIONAL SCIENCE
AND OCCUPATIONAL THERAPY
HEALTH SCIENCE CAMPUS
UNIVERSITY OF SOUTHERN CALIFORNIA
LOS ANGELES, CA 90089

RADHAKRISHNAN, KAVITA, PHD *
ASSOCIATE PROFESSOR
SCHOOL OF NURSING
UNIVERSITY OF TEXAS AUSTIN
AUSTIN, TX 78712

RHEE, KYUNG E, MD
PROFESSOR
DEPARTMENT OF PEDIATRICS
UNIVERSITY OF CALIFORNIA, SAN DIEGO
LA JOLLA, CA 92093

RINI, CHRISTINE, PHD
PROFESSOR
DEPARTMENT OF MEDICAL SOCIAL SCIENCES
FEINBERG SCHOOL OF MEDICINE
NORTHWESTERN UNIVERSITY
CHICAGO, IL 60611

ROPER, JENEVIEVE L, PHD *
ASSISTANT PROFESSOR
LIFE SCIENCES BUILDING
LOYOLA MARYMOUNT UNIVERSITY
LOS ANGELES, CA 90045

SCHENKER, YAEL, MD
PROFESSOR
SECTION OF PALLIATIVE CARE AND MEDICAL ETHICS
DIVISION OF GENERAL INTERNAL MEDICINE
UNIVERSITY OF PITTSBURGH
PITTSBURGH, PA 15213

SEVEN, MEMNUN, PHD *
ASSISTANT PROFESSOR
COLLEGE OF NURSING
UNIVERSITY OF MASSACHUSETTS AMHERST
AMHERST, MA 01003

SMITH, PATRICK J, PHD
ASSOCIATE PROFESSOR
DEPARTMENT OF PSYCHIATRY AND BEHAVIORAL
SCIENCES
MEDICINE AND POPULATION HEALTH SCIENCES
DUKE UNIVERSITY
DURHAM, NC 27710

SUNEJA, GITA, MD *
ASSOCIATE PROFESSOR
DEPARTMENT OF RADIATION ONCOLOGY
SCHOOL OF MEDICINE
UNIVERSITY OF UTAH
SALT LAKE CITY, UT 84112

TAPIA, IGNACIO E, MD *
ASSOCIATE PROFESSOR
DEPARTMENT OF PEDIATRICS
PERELMAN SCHOOL OF MEDICINE
UNIVERSITY OF PENNSYLVANIA
PHILADELPHIA, PA 19104

TEMPLE, JENNIFER L, PHD
PROFESSOR
DEPARTMENTS OF EXERCISE AND NUTRITION SCIENCES
AND COMMUNITY HEALTH AND HEALTH BEHAVIOR
SCHOOL OF PUBLIC HEALTH AND HEALTH PROFESSIONS
UNIVERSITY AT BUFFALO
BUFFALO, NY 14214

UEBELACKER, LISA A, PHD
PROFESSOR
DEPARTMENT OF PSYCHIATRY AND HUMAN BEHAVIOR
BUTLER HOSPITAL
BROWN UNIVERSITY
PROVIDENCE, RI 02906

VAN CLEAVE, JANET H, PHD *
ASSISTANT PROFESSOR
RORY MEYERS COLLEGE OF NURSING
NEW YORK UNIVERSITY
NEW YORK, FL 10010

VISOVSKY, CONSTANCE G, PHD *
ASSOCIATE PROFESSOR
COLLEGE OF NURSING
UNIVERSITY OF SOUTH FLORIDA
TAMPA, FL 33612

VOLERMAN, ANNA, MD *
ASSOCIATE PROFESSOR
DEPARTMENT OF MEDICINE - GENERAL INTERNAL
MEDICINE
THE UNIVERSITY OF CHICAGO
CHICAGO, IL 60637

WEN, KUANG-YI, PHD
ASSOCIATE PROFESSOR
DEPARTMENT OF MEDICAL ONCOLOGY
THOMAS JEFFERSON UNIVERSITY
PHILADELPHIA, PA 19107

SCIENTIFIC REVIEW OFFICER

VOSVICK, MARK A, PHD
SCIENTIFIC REVIEW OFFICER
CENTER FOR SCIENTIFIC REVIEW
NATIONAL INSTITUTES OF HEALTH
BETHESDA, MD 20892

EXTRAMURAL SUPPORT ASSISTANT

WATTS, MELISSA D
EXTRAMURAL SUPPORT ASSISTANT
CENTER FOR SCIENTIFIC REVIEW
NATIONAL INSTITUTE FOR HEALTH
BETHESDA, MD 20892

* Temporary Member. For grant applications, temporary members may participate in the entire meeting or may review only selected applications as needed.

Consultants are required to absent themselves from the room during the review of any application if their presence would constitute or appear to constitute a conflict of interest.
